# Where Is the Artificial Intelligence Applied in Dentistry? Systematic Review and Literature Analysis

**DOI:** 10.3390/healthcare10071269

**Published:** 2022-07-08

**Authors:** Andrej Thurzo, Wanda Urbanová, Bohuslav Novák, Ladislav Czako, Tomáš Siebert, Peter Stano, Simona Mareková, Georgia Fountoulaki, Helena Kosnáčová, Ivan Varga

**Affiliations:** 1 Department of Stomatology and Maxillofacial Surgery, Faculty of Medicine, Comenius University in Bratislava, 81250 Bratislava, Slovakia; bohuslav.novak@fmed.uniba.sk (B.N.); stano28@uniba.sk (P.S.); marekova28@uniba.sk (S.M.); tzina.fountoulaki@gmail.com (G.F.); 2 Department of Simulation and Virtual Medical Education, Faculty of Medicine, Comenius University in Bratislava, Sasinkova 4, 81272 Bratislava, Slovakia; 3 Department of Orthodontics and Cleft Anomalies, Faculty Hospital Kralovske Vinohrady, Dental Clinic 3rd Medical Faculty Charles University, 10034 Prague, Czech Republic; wanda.urbanova@gmail.com; 4 Department of Oral and Maxillofacial Surgery, Faculty of Medicine, Comenius University in Bratislava and University Hospital, 81372 Bratislava, Slovakia; czako@ionline.sk; 5 Department of Stomatology and Maxillofacial Surgery, Jessenius Faculty of Medicine in Martin, Comenius University Bratislava, Kollárova 2, 03659 Martin, Slovakia; tomas.siebert@uniba.sk; 6 Department of Genetics, Cancer Research Institute, Biomedical Research Center, Slovak Academy of Sciences, Dubravska Cesta 9, 84505 Bratislava, Slovakia; 7 Department of Histology and Embryology, Faculty of Medicine, Comenius University in Bratislava, 81372 Bratislava, Slovakia; ivan.varga@fmed.uniba.sk

**Keywords:** artificial intelligence, deep learning, dentistry, orthodontics, maxillofacial surgery, endodontics, periodontics, prosthodontics, forensic odontology, evidence-based practice

## Abstract

This literature research had two main objectives. The first objective was to quantify how frequently artificial intelligence (AI) was utilized in dental literature from 2011 until 2021. The second objective was to distinguish the focus of such publications; in particular, dental field and topic. The main inclusion criterium was an original article or review in English focused on dental utilization of AI. All other types of publications or non-dental or non-AI-focused were excluded. The information sources were Web of Science, PubMed, Scopus, and Google Scholar, queried on 19 April 2022. The search string was “artificial intelligence” AND (dental OR dentistry OR tooth OR teeth OR dentofacial OR maxillofacial OR orofacial OR orthodontics OR endodontics OR periodontics OR prosthodontics). Following the removal of duplicates, all remaining publications were returned by searches and were screened by three independent operators to minimize the risk of bias. The analysis of 2011–2021 publications identified 4413 records, from which 1497 were finally selected and calculated according to the year of publication. The results confirmed a historically unprecedented boom in AI dental publications, with an average increase of 21.6% per year over the last decade and a 34.9% increase per year over the last 5 years. In the achievement of the second objective, qualitative assessment of dental AI publications since 2021 identified 1717 records, with 497 papers finally selected. The results of this assessment indicated the relative proportions of focal topics, as follows: radiology 26.36%, orthodontics 18.31%, general scope 17.10%, restorative 12.09%, surgery 11.87% and education 5.63%. The review confirms that the current use of artificial intelligence in dentistry is concentrated mainly around the evaluation of digital diagnostic methods, especially radiology; however, its implementation is expected to gradually penetrate all parts of the profession.

## 1. Introduction

A brief history of artificial intelligence (AI) and the current context of its dental applications must be introduced here to facilitate understanding of the outcomes of the literature review.

**Figure 1:**
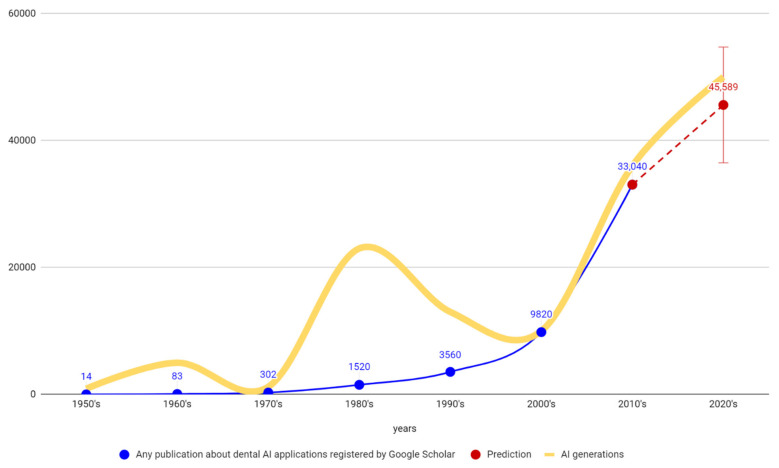
Visualization of 1st, 2nd and current 3rd waves of AI generations (booms) in human history does not correspond to the history of Google Scholar dental-AI publications, thus indicating that only the current 3rd AI generation is mature enough to offer undisputed potential for research and application in dentistry. Visualized prediction for the next decade of dental AI publications was calculated by simple extrapolation using a linear regression, and is just for illustration.

The term “artificial intelligence” (AI), defined as computerized synthetic human cognitive function, was first introduced in 1956 at Dartmouth University [1]. Since then, AI has been exponentially expanding in all fields [2,3]. There were three booms and two cooling periods in its utilization (Figure 1). These technological AI waves do not correspond to many dental publications regarding AI in dentistry. This may indicate that only the recent 3rd AI generation represents a mature technology with clinical impacts in dentistry. All modern progress and innovations in dentistry have been achieved due to the scientific approach that is currently experiencing a paradigm shift in the interdisciplinary relationship between AI and data science [4,5]. The recent boom is powered by major AI subfields, which include:

1. Machine Learning (ML)
2. Neural Networks (ANN)
3. Computation
4. Vision, Robotics
5. Expert Systems
6. Speech Processing
7. Natural Language Processing

**Figure 2:**
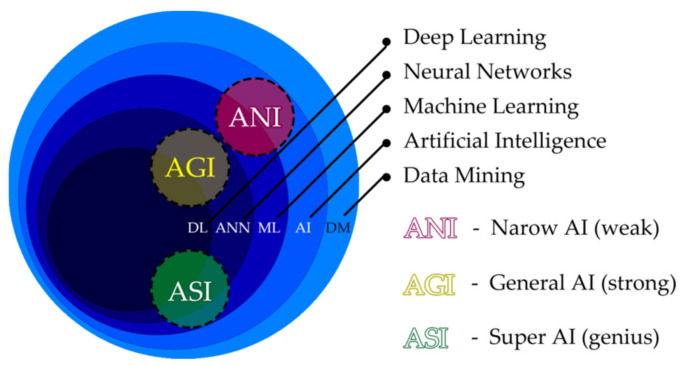
General AI categorization defines three types: artificial narrow intelligence (ANI), artificial general intelligence (AGI), and artificial super intelligence (ASI).

To understand the current and future clinical AI utilizations in dentistry, we must first comprehend the elemental AI technologies. Figure 2 represents the relationship between the various technologies [6,7,8,9]. AI connotes fundamental technologies including machine learning, artificial neural networks (ANN), and deep learning. AIs are commonly categorized into three types: artificial narrow intelligence (ANI), artificial general intelligence (AGI), and artificial super intelligence (ASI). ANI, known as weak AI, possesses narrow abilities suitable for very specific tasks. These systems do not perform outside the single task for which they are designed. For clinical purposes in dentistry (e.g., cone beam computed tomography (CBCT), 3D convolutional neural networks (3D CNN)) are more suitable for more complex AI implementations. These are frequently applied even in interdisciplinary fields, such as forensic dentistry [10,11]. AGI known as “strong” or “deep AI” is about as capable of solving problems as a human. We as clinicians have the abilities of abstract thinking, strategizing and producing creative ideas, which remain weak areas for most current advanced AI algorithms [6]. AGI will be able to think very similarly to a human. ASI will exceed human capabilities and will be able to learn and improve itself beyond our comprehension.

Controversies regarding AI application include a noteworthy paradox regarding legal liability for AI algorithm assessment errors, and thus possible incorrect medical intervention. As we know, AI can be trained as according to the biases of any person [12]. Another paradoxical situation addressed by Zhang et al. [13] is that with deep AI, we have no understanding of how the algorithm obtained the result. Therefore, recent research has been conducted into “explainable artificial intelligence (XAI) to overcome this limitation of the black-box nature of AI processes. Compared with AI techniques such as deep learning, XAI can provide both decision-making and explanations of the model” (Zhang et al. [13]). Various publications have been confirmed as addressing this topic [14,15].

For clinical AI applications in dentistry, a full digital workflow transformation is necessary [16,17,18,19,20,21]. Currently, various examples of advanced dental technologies can be found based on digital workflows [7,16,19,20,21,22,23,24,25,26,27,28,29,30]. Their goal is to enhance the efficiency and quality of delivered services. AI implementations are invisible algorithms in software tools in the majority of these processes [5,6,31,32,33,34,35,36,37]. They often overlap various dental specialties and categorizations. Currently, they mostly include:

1. AI in X-ray and other diagnostics, caries [29,30,31,36,37,38,39,40,41,42,43,44]
2. AI in implant dentistry [26,33,45,46]
3. AI in photography analysis [27,28,29,47]
4. AI in practice management, tele-dentistry, patient coaching [44,48,49,50,51,52,53,54]
5. AI in clinical predictions (virtual simulation, aging, growth) [5,55,56,57,58,59]

In the context of other literature reviews, for the first 109 days of the year 2022, Google Scholar was already registering 304 articles focused on AI utilization in dentistry, among which 10 were common reviews (with the search term only in the article title). However, these dental publications seldom covered the AI implementation in detail. When they covered it, most of them only mentioned it in the Section 4 without actually investigating it. After refinement of the results, only eight were comparable to this paper with comprehensive focus on dentistry including literature analysis, although these were either missing trend analysis or had a scope of no longer than 2 years. Of these eight reviews only two were registered in SCOPUS/Web of Science [7,60].

Scopus published 35 articles regarding dental AI applications in the year 2022. Only six were reviews fully overlapping with the Web of Science query results. However, there was one interesting comprehensive review by Carrillo-Perez et al. [7]. This study was published in January 2022, although with early access in November 2021, and it was indexed in Web of Science in December 2021, which explains why it was not provided as a result of the original query for Web of Science 2022 publications. This comprehensive review of the use of AI in dentistry devotes special attention to the area of esthetic dentistry and color research, although it covers the whole dental field. Research of the MEDLINE/PubMed, Web of Science, and Scopus databases for papers published in the English language in the last 20 years on AI decision-support systems identified an increasing trend of AI impacts on the dental profession. The most highly cited review papers on this topic are from year 2021, when Shan et al. [61] presented the progress and potential dental applications of AI in medical-aided diagnosis, treatment, and disease prediction.

Recently, various valuable narrative reviews were published that concluded that the AI approach to imaging technology is profoundly changing the established protocols of diagnosis in dentistry [4,5,26,62,63,64]. The application of AI in healthcare is an area of significant attention and importance. There are unusually strong drivers to accelerate the implementation of innovative AI interventions, which may not be supported by the available evidence, and for which the usual systems of appraisal may not yet be sufficient. This is the reason why various reporting guidelines of AI interventions were recently introduced [65,66,67,68].

Regarding the rationale, context, and significance of this review and literature analysis, the main purpose was to provide an unbiased outline of how much attention AI utilization in dental research has received over the years (2011–2021), and on what workflows it is currently focused (qualitative assessment of last 15 months).

An assessment of the current proportions of AI focus in particular dental fields or topics with qualitative analysis of literature is undertaken on unprecedented scale in this paper. This systematic review is one of the most comprehensive regarding AI utilization in dentistry. As an outcome of the literature analysis, the paper also briefly elaborates on the most prominent uses of AI in each dental specialization. These excerpts from clinical AI publications indicate where AI is already clinically implemented in dentistry.

This paper’s systematic review followed two objectives:

1. To quantify how frequently artificial intelligence (AI) was utilized in dental literature for every year from 2011 until 2021.
2. To identify the focus of such publications with regard to particular dental fields and topics from January 2021 until the present.

The literature analysis of this paper presents independent research of current clinical publications and provides an extract of the most frequent clinical scenarios in dentistry where AI is currently implemented. The outcome of this analysis is a brief excerpt for each dental specialty, which may be valuable for clinicians. The specialty topics are:

1. Orthodontics
2. Oral and maxillofacial surgery
3. Therapeutic Dentistry
4. Periodontology
5. Endodontics
6. Craniofacial disorders including cleft management
7. Prosthodontics and smile design
8. Airway management

From a general perspective, this paper provides a broad picture of the current AI-induced paradigm shift in dentistry, which many dental professionals have not yet fully grasped. Better understanding of these changes in the dental community may facilitate more rapid adoption of AI tools, which will lead not only to better accuracy and effectiveness of treatment, but also to greater affordability of dental treatments.

## 2. Materials and Methods

### 2.1. The Framework, the Protocol and Research Questions

This paper presents a literature analysis and a systematic review registered with the international prospective register of systematic reviews—PROSPERO—as “CRD42022327520” and is also registered with the International Registry of Systematic Reviews/Meta-Analyses as “reviewregistry1349”. PRISM protocols are available.

This systematic review has no meta-analysis, as there was no difficult quantitative estimation to be calculated for the effect of any treatment intervention or exposure. This systematic review is not a Cochrane review, as there was no aim to evaluate any treatment intervention; however, it complies with the Cochrane guidelines, including updated PRISM protocols.

The questions of the review are simple and clearly defined, as presented in the protocols of the review. This systematic review answers defined research questions by collecting and summarizing all empirical evidence that fits the pre-specified eligibility criteria.

This review is intended for the practitioners of the dental community who prefer evidence-based practice (EBP). A specialized framework called PICO was implemented to form the questions and facilitate the literature research.

Systematic review questions were focused on narrow parameters. The paper follows the newest updated guideline from the Preferred Reporting Items for Systematic Reviews (PRISMA) 2020 statement [69]. The study was designed according to the updated PRISMA 2020 protocol checklist, and is registered in two mentioned international online portals for systematic review registrations. 

The full review protocol is publicly available. PRISMA 2020 checklists and queried data are available as Supplementary Materials.

Research questions:

1. What is the quantity per year of publications focused on dental AI utilization? Is it increasing? Identify the annual publication count. (2011–2021)
2. What are the qualitative characteristics focused on and the proportion of focus devoted to particular dental fields and specialized topics in recent dental AI publications since 2021?

This paper performs two kinds of analysis:

1. Quantitative assessment of dental AI publications in the past decade.
2. Qualitative assessment of the current literature from 2021 until the present.

The methods of this systematic review build on the newest protocol and guidelines for systematic reviews from PRISMA-P, published in 2015, aiming to facilitate the development and reporting of systematic review protocols [70,71]. The protocol is detailed in Table 1, which describes the methods of this systematic review. Except for the information in Table 1, exclusion criteria included irrelevance (e.g., articles retrieved because of a coincidental abbreviation “AI—Amelogenesis Imperfecta”), or other, mostly technical articles that were non-dental or not written in English.

### 2.2. Evaluating the Past Publications 2011–2021

On 19 April 2022, PubMed, Scopus, Web of Science—Core Collection, and Google Scholar were queried. The focus was on publications from between 2011 and 2021. The Boolean logic of the search is detailed in Table 1 for each of the four electronic databases.

To allow batch export, all four literature databases were queried with a software called “Publish or Perish” (https://harzing.com/resources/publish-or-perish (accessed on 23 March 2022) [73]). Microsoft Windows version used: 8.2.3944 (from 23 March 2022). In this program, access to Scopus and PubMed was managed using registered keys generated for the application programming interface (API). Access to the Web of Science (WOS) was solved via a IP login/password assigned to Comenius University in Bratislava. Google Scholar (GS) access was obtained without any obstacles.

### 2.3. Evaluating the Present Publications 2011–2021

On 19 April 2022, we queried the PubMed, Scopus, Web of Science—Core Collection, and Google Scholar services to inquire about the recent articles and reviews published between 1 January 2021 and 19 April 2022, with the objective of quantitative and qualitative assessment of relevant publications. Table 1 describes all the review protocol details.

## 3. Results

### 3.1. The Past Publications 2011–2021

All four literature databases searched all fields of the publications (i.e., not limited to title, abstract, and keywords). Google Scholar yielded far more publications than any other database. This is often explained by the fact that it has the broadest literature coverage, as well as by its ability to gain access to the full text of the publications, which is a typical finding in literature analysis publications [74]. Google Scholar does not have a setting that allows restriction of the results to peer-reviewed articles only. As Google Scholar (GS) yielded such large numbers of publications that individual human verification was impossible, the results of the Google Scholar query were processed with a stricter query (Table 1). In GS, the citations and patents were not searched. Citation searching is important to identify all included studies. These are essential if systematic reviews are to avoid producing biased conclusions. Citation searching in WOS was more efficient than in GS, which required additional time to remove non-English language studies. This is the reason the GS citation search was finally not enabled. The recent evaluation by Cantrell et al. [75] of supplementary search methods, including citation searching, showed that little evidence exists on methods for prioritizing databases for citation searching or for establishing whether using multiple sources is beneficial.

All publications returned by the searches were initially included. The content of each one was manually checked, and a publication was excluded if it was irrelevant (e.g., dealing with non-dental AI utilization or using AI as abbreviation for Amelogenesis Imperfecta) or not written in English. This literature survey excluded books, dissertations, patents, and citations resulting from the search.

**Figure 3:**
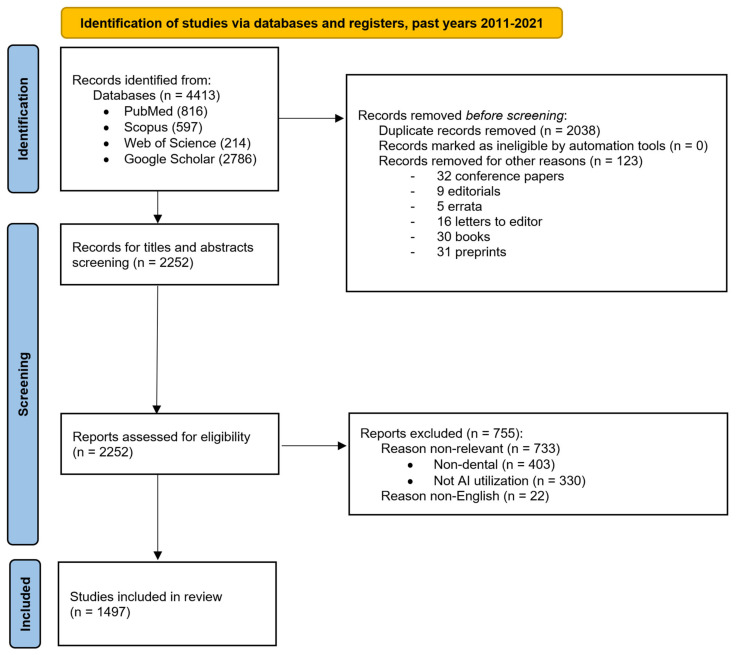
Objective 1: the number of dental publications focused on AI utilization. The PRISMA flow chart diagram depicts the flow of information through the different phases of the systematic review for the first objective.

The literature screening process is illustrated Figure 3 in the PRISMA flow chart diagram, showing the flow of information through the different phases of the systematic review. It maps out the number of records identified, included, and excluded, and the reasons for exclusions.

**Figure 4:**
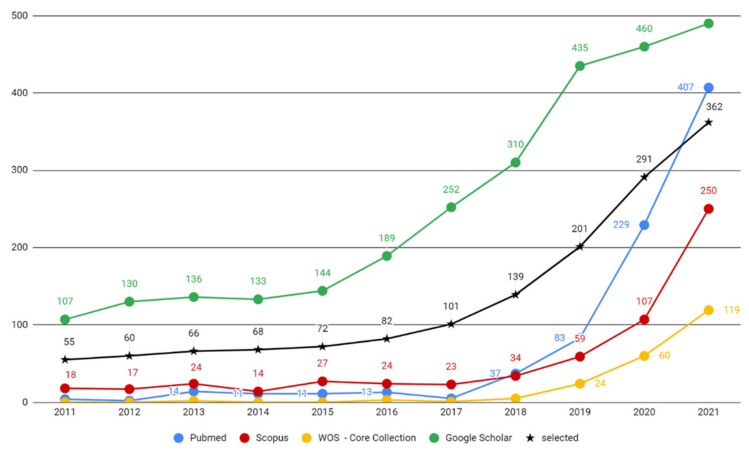
Annual counts of AI-focused dental publications (2011–2021).

Of 4413 registered records, 2252 were screened and 1497 were included to accomplish the first objective of this systematic review. After distribution of the included studies into publication years, Figure 4 visualizes the counts of originally selected and finally included papers, and their increase over the years.

Table 2 visualizes the annual publication counts from electronic databases PubMed, Scopus, Web of Science—Core Collection, and Google Scholar, contrasting the registered publications with the publications finally included, with the percentual annual increase noted.

### 3.2. The Current Publications 2011–2021 Analysis

**Figure 5:**
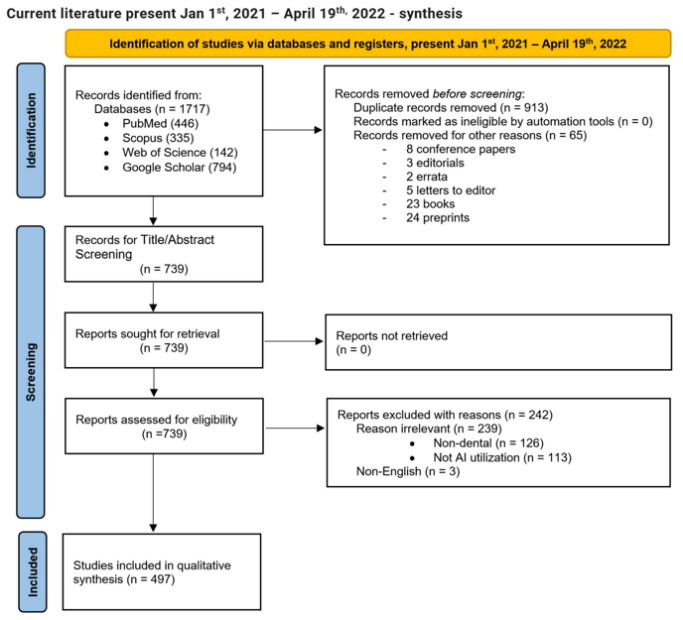
The PRISMA flow chart diagram depicts the flow of information through the different phases of a systematic review. It maps out the number of records identified, included, and excluded, and the reasons for exclusions. Different templates are available depending on the type of review (new or updated) and sources used to identify studies.

On 19 April 2022, PubMed, Scopus, Web of Science—Core Collection, and Google Scholar were queried. The focus was on recent articles and reviews published between 1 January 2021 and 19 April 2022, with the objective of quantitative and qualitative assessment of relevant publications. Figure 5 shows the flow of information through the different phases of this systematic review for the second objective. It maps out the number of records identified, included, and excluded, and the reasons for exclusions.

**Figure 6:**
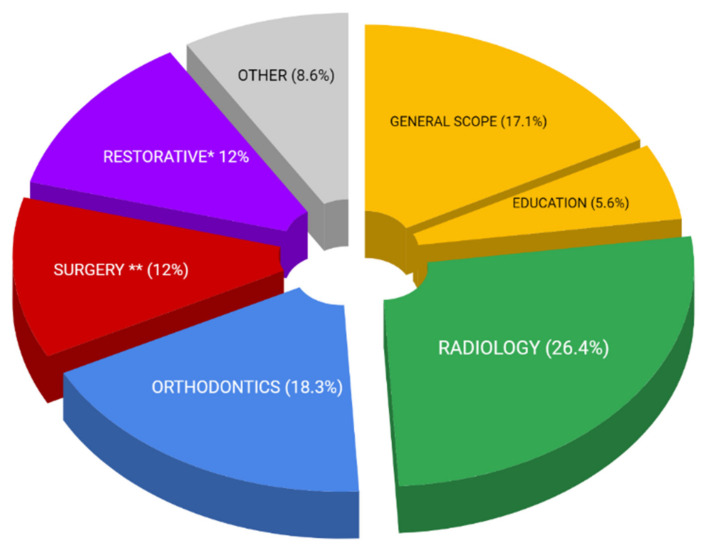
Simplified pie graph visualizing proportionality of current AI focus in dentistry (1 January 2021–19 April 2022), where the focus on implants and cancer treatment is covered by Surgery, and Restorative dentistry also covers Endodontics and Prosthodontics. * Restorative dentistry in this chart covers also Endodontics and Pediatric dentistry. ** Surgery represents also Cancer diagnostics and Dental implantology.

**Figure 7:**
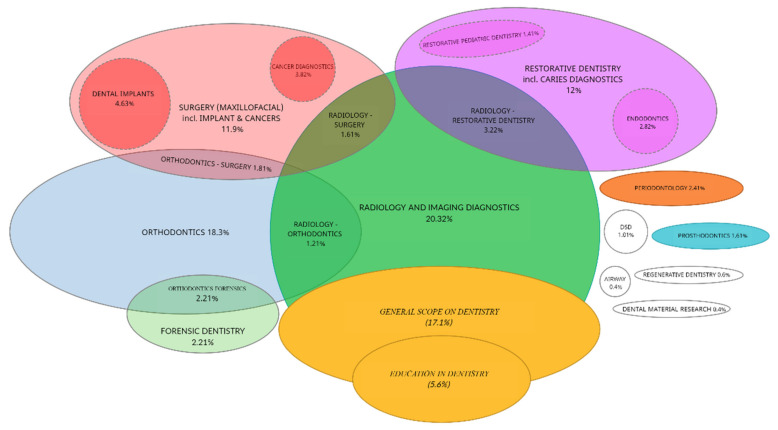
Venn diagram illustrating where dental AI use is currently focused, with relationships among the groups.

Table 3 shows the results. Of the 1717 registered records, 739 were screened and 497 were included in accomplishing the second objective of this systematic review. After assessment, 362 publications were included for the year 2021, and 135 studies were included for the year 2022, as shown in Table 3. All 497 studies were qualitatively assigned to one of the 22 focus groups listed in Table 1. After qualitative assessment of these publications from the last 474 days (1 January 2021–19 April 2022), two data visualizations were made:

1. Simplified proportionality pie graph (Figure 6).
2. Venn diagram to illustrate the relationships among the groups (Figure 7).

These data visualizations are an important contribution to understanding of the current focus of AI applications in dentistry. Both visualizations are based on data shown in Table 4.

For a better overview of the topic, Table 5 presents the ten most impactful AI publications in dentistry according to their total and average citations per year.

### 3.3. Literature Analysis of Current AI Applications in Orthodontics

AI is undoubtedly shifting the paradigm in orthodontics. Orthodontics may now belong among the dental specialties most affected by AI. Cephalometric analysis—a cornerstone of treatment planning—now utilizes the power of automated CBCT segmentations with automated 3D cephalometric analysis based on advanced 3D CNN algorithms [85]. Of all skeletal and soft-tissue structures available for orthodontic diagnostics, the face is among the most important, albeit the most difficult for humans to scientifically comprehend [30]. Now AI automated facial surface analysis sourcing from CBCT, Lidar from ubiquitous smartphones, or any other face scanner can be utilized for increased diagnostic precision and efficiency [44,59,86,87,88]. Facial growth and aging predictions represent another specialty leaving the human domain as AI algorithms are taking over prediction and planning [55,89,90]. Various companies have spent the last decade gathering big data regarding teeth movement resulting from aligner treatments, which have provided them with an essential foundation for advanced AI implementations supporting effective teeth movement. However, other major aspects of orthodontics—patient communication, coaching, and no less important, clinician control over treatment progress, have become a new domain for AI implementations [49,91,92,93,94,95,96]. Many of these implementations were introduced as telehealth solutions, benefiting from the interpersonal contact restrictions of the COVID19 pandemic. Some of them have even provided a useful AI-managed tool for the orthodontic retention phase or pre-treatment phase, so that clinical conditions, defined by orthodontic specialists, could be regularly evaluated by AI through patients’ home-video recordings [93,94]. The aspect of the orthodontic specialty that is not significantly affected by AI is customized device designing and manufacturing. AI processes are well-implemented in clear-aligner mass production [97,98,99,100], although artificial intelligence played a significant role in safely handling pandemic challenges in orthodontic care and education [101].

### 3.4. Literature Analysis of Current AI Applications in Oral and Maxillofacial Surgery

In combination with human skills, AI promises to be the most useful and valuable tool in the everyday practice of oral and maxillofacial surgery [102], similarly to modern medical imaging methods, such as variations based on computed tomography (CT), CBCT, and magnetic resonance imaging (MRI), which have transformed how clinicians visualize facial anatomy and pathology [103]. Representative articles are already reporting on AI algorithms aiding in diagnosis, therapeutic decision making, preoperative planning, and prediction and evaluation of outcomes. Thanks to their advanced classification, learning, prediction, and detection capabilities, AI algorithms complement human skills while minimizing their imperfections and inaccuracies [4]. Several of the registered studies reported preference for the use of AI in the detection of head and neck tumors using image data (radiographic, microscopic and ultrasonographic images) [2,23,60,104,105]. The main goal of these papers was to apply a hybrid of feature-selection and machine-learning methods in oral cancer prognosis based on the parameters in correlation with clinicopathologic and genomic markers. The term “radiomics” refers to the analysis and extraction of quantities of data from imaging features generated by CT, positron emission tomography (PET/CT), and MRI [106]. Other applications for AI in maxillofacial surgery include predicting results and planning orthognathic and craniofacial surgical procedures (i.e., after skeletal trauma) with the use of digital imaging, photographs [107], 3D photography and intraoral scans. The tissue-prediction field needs more relevant 3D data and outputs, as the currently available data are a combination of different measurements, 2D and 3D planning, and imaging [48]. Significant investments are also being made by the industry in the research and development of applied AI to digital orthodontics and robotic surgery [4,108]. AI has the potential to be an immensely powerful tool in oral and maxillofacial surgery in the early future, and it is the responsibility of all maxillofacial surgeons to collect virtual data and to achieve a positive symbiosis with the clinical use of AI.

### 3.5. Literature Analysis of Current AI Applications in Therapeutic Dentistry

Therapeutic dentistry takes advantage of AI in different fields, mainly in image-based automatic detection of diseases and other diagnosis-support systems [76]. Detecting the dental caries in periapical radiographs can be performed with the help of a deep-learning-based CNN (convolutional neural network) algorithm [31,32,79,109,110,111]. This can be useful to enhance dental caries detection and diagnosis in clinical dental practice [76]. AI can also support proximal caries detection on bitewings. This could be cost- effective, due to its higher sensitivity in the detection and arrest of early caries lesions [112,113]. CNN-based AI algorithms can be beneficial to the dentist as clinical decision support systems [114]. A deep CNN system can be used to number teeth in bitewing radiographs and save the dentist time by automatically preparing dental charts [115]. AI models may serve as powerful tools in the diagnosis of dental caries. Further studies are required to evaluate the clinical performance of AI models [22]. A common problem for patients is tooth surface loss (TSL), which involves an irreversible, multifactorial, physiologic, pathologic, or functional loss of hard dental tissue [116]. ANN can be used for the prediction of TSL with a reasonable degree of accuracy [117].

### 3.6. Literature Analysis of Current AI Applications in Periodontology

Periodontal health is represented by the stability, aesthetics, and function of the teeth as a factor in social quality of life. Periodontitis is a major public health problem due to its high prevalence and because it can lead to tooth loss and later to the loss of all teeth. AI has received enormous attention and has gone through a transition stage from being a purely statistical tool to being one of the main drivers of modern medicine [118]. AI software was designed with automated search algorithms with increased capability to extract, analyze, and correlate large quantities of relevant, multifactorial complications, as well as sub-sorting the predisposing factors of any investigated condition [119]. The use of AI techniques in combination with different data types for the diagnosis of periodontal diseases has been extensively explored in literature (W. P. Chen et al., 2018; Farhadian et al., 2020), [120,121]. The prevention of oral diseases based on AI impacts on oral hygiene behavior has also been reported. A novel method, based on wrist-worn inertial sensors to detect brushing and flossing behaviors, was proposed [7,122,123].

### 3.7. Literature Analysis of Current AI Applications in Endodontics

The goal of endodontic treatment is to provide the best-quality treatment with the intention of retaining the tooth in its functional state and preventing any further complications [124,125]. AI models have demonstrated various applications in endodontics, such as studying root canal system anatomy, detecting periapical lesions and root fractures, determining working length measurements, predicting the viability of dental pulp stem cells, and predicting the success of retreatment procedures [126]. Computer-aided diagnosis (CAD) has mainly focused on the development of artificial intelligence for the assessment of periapical lesions using digital periapical radiographs, panoramic radiographs, and CBCT images [78,125]. AI-based models are very efficient in determining the apical foramen and working length. These models can be of great assistance for less experienced dentists and non-specialists, as they can be used in clinical applications [118,125]. Artificial intelligence technology is widely used in the diagnosis (radiographic detection, CBCT images) of periapical pathologies, demonstrating satisfactory results with high sensitivity and moderate specificity [127,128,129]. It is especially important to detect vertical root fractures (VRFs) at an early stage to prevent damage to supporting structures. AI technologies proved to be very efficient in comparison to periapical radiographs in diagnosing VRFs on CBCT images [130,131].

### 3.8. Literature Analysis of Current AI Applications in Patients with Cleft Lip and Palate and in Genetic Syndromes with Orofacial Features

In patients with cleft lip and palate (CLP), AI is applied in estimating risk factors, prenatal and postnatal diagnosis, growth predictions, and various treatment strategies. Most of these artificial intelligence models are based on artificial neural networks.

AI can estimate the occurrence of CLP, thanks to predictive algorithms [132], and can identify environmental and genetic risk factors in different populations [133,134]. Whilst cleft lip is unmissable in a fetal ultrasound examination, diagnosis of cleft palate in prenatal stages is difficult. AI might be helpful in the form of the syntactic pattern recognition method developed by Jurek et al. [135]. Postnatal detection and classification of unilateral cleft alveolus with or without cleft palate on panoramic radiographs can be performed using a deep learning system invented by Kuwada et al. [136]. Zhang et al. [137] estimated the cleft volume before secondary alveolar bone grafting with the help of a neural network, using a volumetric registration-based framework to reconstruct the defect and estimate its volume. Determining and comparing orthodontic conditions in patients with CLP by modeling the similarity function may be helpful in future while planning orthodontic treatment [138]. AI technology has been also applied in patients with CLP for identifying cephalometric landmarks [139], evaluating cephalometric analysis [140], predicting the need for orthognathic surgery after the end of growth [141] and predicting soft tissue changes after surgery [142]. Anthropometric analysis of facial symmetry in photographs of CLP patients can be conducted with a specialized computer system called “AID”, which has proved to be simple, time-efficient, and reliable for this task [143]. Using AI to score the facial attractiveness of treated CLP patients at the end of treatment can eliminate dispersion-related issues when panel ratings are performed [144]. Speech assessment in patients with CLP can be conducted with the help of AI, mostly concentrating on the presence and severity of hypernasality [145,146].

### 3.9. Literature Analysis of Current AI Applications in Prosthodontics & Smile Design

With the emergence of the first systems based on artificial intelligence in prosthodontics as a well-defined dental discipline, the paradigm shift has demonstrated itself already in its applications in automated diagnostics, as a predictive measure, and as a classification or identification tool [147]. Prosthodontics today make use of every aspect of digital dentistry. Workflows that once started with analogue impressions now start with an intraoral scan. Intraoral scanners have proven to be accurate enough for daily practice, especially in single-crown or smaller bridge restorations [148,149,150]. AI is used during the scanning process to automatically remove excess soft tissues and material [151,152]. The next step after intraoral scan acquisition in fixed prosthodontics is margin detection—AI is used for this purpose, and the proposed margin can be modified afterwards [153,154,155]. Computer-aided design/computer-aided manufacturing (CAD/CAM) is used in the design and manufacturing of fixed and removable dental restorations. In this process, machine learning can use millions of natural crowns to create the best possible crown design for certain situations [156,157,158]. Artificial intelligence can also be used to predict debonding of CAD/CAM restorations based on die images [159]. In removable prosthodontics, dental arches can be classified with the use of convolutional neural network (CNN) [160,161]. In edentulous patients, it is always a challenge for the dental technician to set up denture teeth to meet both functional and aesthetic requirements. Machine learning in CAD/CAM software can recreate acceptable intermaxillary relations by setting teeth up correctly [162]. In complicated aesthetic cases that involve a single central incisor or multiple frontal teeth, AI can help with precise shade matching [163]. In implant prosthodontics, intraoral scanners can recognize implant locations and directly import them in CAD software [26,33,164]. AI can also provide optimization of dental implant design, but it still needs some adjustments [26,33,45,46,165]. Smile designing is currently a popular part of digital workflow that intersects across various dental disciplines. It takes advantage of the unprecedented accessibility of digital scanning, including 3D face scanning and the availability of virtual fusions of 3D data, such as segmented CBCT, intraoral scans, and face scans, resulting in the virtualization of patient morphology, which is the cornerstone of any treatment planning affecting the patient’s smile [166,167,168,169]. Smile designing started with simple drawings on paper using printed 2D photographs of the patients [170]. It later developed towards presenting software, such as PowerPoint/Keynote, and subsequently dedicated software was programmed. Because of its benefits, various manufacturers provide software that uses AI to facilitate the smile-designing process [171,172,173].

### 3.10. Literature Analysis of Current AI Applications in Airway Management

AI is the simulation of human intelligence by computer systems and encompasses reasoning, learning, processing, and display of information [174]. A subset of AI is machine learning, which is divided into 2 main types: supervised and unsupervised, defined by the presence or absence of a data guidance basis [175,176]. The increase in healthcare data will profoundly change the nature of medical care. Smartwatches are widely used as fitness trackers but also as a diagnostic tool in sleep medicine. Their hardware is designed to collect data and monitor the duration of sleep, the changes in the level of oxygen, blood pressure, heart beats, and fluctuations during sleep, making them able to detect atrial fibrillation and sleep apnea [177]. Smartwatch-based systems, such as ApneaDetector, exploit built-in sensors in smartwatches to obtain audio data exported from noisy and multi-axis sensing data assisting in diagnosis of sleep apnea [178]. AI has also been used in pediatric anesthesia airway management, assisting pediatric patients with endotracheal intubation in the form of SmartScope. The machine-learning-based algorithm that Matava C. et al. developed [179] can identify the position of the vocal cords and the airway/tracheal anatomy, helping with confirmation of intubation using bronchoscopy and video-laryngoscopy. As the study mentions, the localization of the tracheal rings is possible using segmentation module output as a tracheal GPS. AI can also assist physicians during the diagnosis of respiratory disorders, such as chronic obstructive pulmonary disease (COPD, asthma, and even lung cancer, by collecting data such as patient history, imaging (Ct scans, X-rays, bronchoscopy), and data from physical examination [175,176,180,181,182,183,184,185,186,187,188,189,190].

## 4. Discussion

The results indicate that the boom in AI utilization in dentistry is accelerating. All the necessary requirements are met, including digital workflows with digital big data, which are now available to train advanced neural networks. The opinions of many clinicians towards AI utilization in dentistry are still burdened with skepticism; they view it as potentially a hyped trend and consider AI as merely a trending buzzword with questionable credibility and depleted potential for real contributions to the future development of the dental field. The objective identification of the high quantity of dental research utilizing AI is an unbiased indicator of the actual potential for AI implementation, and the long-term evaluation answers important questions regarding the true significance of AI for the dental field. The paper answers the question of “Where in dentistry is the AI currently truly applied?”.

Results from the first objective of this paper reveal a stable increasing trend with insignificant stagnation in the years 2013–2014, and sudden escalation from the year 2015 (Figure 5). The graph in this figure also indicates an interesting phenomenon: that Google Scholar was the earliest to recognize the rising publication trend, and was followed by other digital databases. This might indicate a useful predictive feature of the Google Scholar database. Results also reveal an unprecedented boost of publications in Scopus, WoS and PubMed in the years 2019–2021, which might imply that the COVID-19 pandemic served as an accelerator in the implementation of AI-powered technologies in dentistry. The pandemic situation may have forced an accelerated utilization of the smart technologies that are often combined with tele-health solutions [7,60]. Current high-quality publications regarding AI utilization in the category of Dentistry, Oral Surgery & Medicine registered by Web of Science—Core Collection include 24 review articles in the year 2021 and 5 review articles published in the year 2022 [60,191,192,193]. Of these, only one paper covered the whole dental field. Kishimoto et al. [60] published a literature review of PubMed, Cochrane Library, and Scopus articles, focusing on the AI architecture types in 58 selected publications out of 422 studies from 1996 to 2019. Approximately half of them (29/58) employed neural networks. The most cited paper from Khanagar et al. [194] with their systematic review concluded that “the performance of AI based automated systems is frequently excellent and that they frequently mimic the precision and accuracy of well-trained specialists” [194]. It also stated that “in some studies it was found that these systems were even able to outmatch dental specialists in terms of performance and accuracy” [194]. For better comprehension of the power of the current dynamics in dental AI publications, it might be useful to consider that the sum of these publications from the last two years outmatches all such publications from the last two decades (according to Google Scholar). Results from the first objective of this paper shown in Table 2 confirm a steady increase with an unprecedent acceleration during the last five years. This confirms the working hypothesis of this paper.

The second objective of this paper was to identify where the utilization of AI is currently focused in dentistry. The visualization of the results in Figure 6 with a simplified pie graph reveal that the majority of AI utilization is currently focused on and is also sourced from Dental radiology. From the advances in 3D image processing with advanced AI algorithms, the benefits have spilled over into other dental specialties, such as orthodontics, maxillo-facial surgery treatment planning, or restorative dentistry, including caries diagnostics. The results of the second objective of this paper, visualized by means of the Venn diagrams in Figure 7, show that a significant proportion of dental AI utilization is not focused on any particular dental field (17.1%). AI is most often implemented in orthodontics, especially in treatment planning, where automation for CBCT segmentation and 3D cephalometric analysis based on advanced 3D CNN algorithms is used [30,44,55,59,85,86,87,88,89,90]. It is also possible to predict aging and facial growth by AI prediction and planning. Overall care of the orthodontic patient, from determining the movement of the teeth to communication, guidance, and telemedicine, is possible using AI [49,91,92,93,94,95]. The literature research also showed that the scale of AI-affected orthodontic subfields is extensive, ranging from pre-treatment AI monitoring, through AI growth/aging 3D morphological predictions and virtual treatment planning [59,85,103,195] with later in-treatment decision-making, monitoring, and patient management, to post-treatment evaluation and retention monitoring [196,197]. In-office treatment personalization by means of 3D printed customized devices easily designed with the support of AI is possible in the future with current AI implementation trends, although this is yet to be realized [97,98,99,100].

There were 14 systematic reviews published in the last two years focused on AI in dentistry [119,198,199,200,201]. Only three of them were focused on dentistry with a general scope. In the first review, Khanagar et al. [194] conclude upon the analysis of 43 selected papers that the majority of the documented work was focused on AI models relying on convolutional neural networks (CNNs) and ANNs. Their review also concludes that AI-based automated systems frequently mimicked the precision and accuracy of trained specialists, and in some studies, it was found that these systems were even able to outmatch dental specialists. The second systematic review, by Ahmed et al. [119], analyzes 38 articles and comes to similar conclusions about the current utilization advantages and the future potential of AI in dentistry. The third systematic review, from Farook et al. [201], evaluates machine learning with a broad dental and orofacial healthcare scope with regard to diagnosing diseases with symptomatic pain.

Cochrane library provided only two results of AI in dentistry. Both were trials from 2021; one was a comparative study of dentists’ ability to detect caries in bitewings with and without AI support [202], and the second clinical trial focused on management of edentulous patients with AI utilization [203].

In conclusion, this review of the literature provided a practical view of AI-powered practices in every dental specialty researched, proving that “modus operandi” has been already impacted by AI, and an AI-driven paradigm shift is in progress. In correlation with this conclusion, the systematic review presented in this paper supported this conclusion with more methodical scope on the subspecialities of dentistry, revealing that the majority of the AI progress in dentistry is emerging from digital radio-diagnostic and other diagnostic AI-processed technologies.

The results of the analysis of 1497 studies published in 2011–2021 confirmed unprecedented annual growth in dental publications focusing on AI, accelerating since 2015. The annual number has grown with an average increase of 21.6% per year over the last decade, and a 34.9% increase per year over the last 5 years. This contrasts with the average “one-digit” annual growth typical of the beginning of this century.

The dramatic increase in publications in recent years confirms the true clinical value of AI implementation in dentistry, which may lead us to change our basic assumptions.

The results of an analysis of current studies published between 1 January 2021 and 19 April 2022, totaling 497 studies selected from 1717 identified records, revealed the major areas in which AI is currently applied in dental publications. Naturally, dental radiology (26.36%), which represents every fourth publication, is the leading specialization in the field of dentistry for the utilization of AI. AI use in diagnostics overlapped among dental specializations, occurring in the fields of orthodontics (18.31%), restorative dentistry (12.09%), and maxillo-facial surgery (11.87%).

The publications on AI utilization addressed parts of the workflow that were common across all dental specializations, including diagnostics, treatment planning, X-ray processing, patient communication, and evaluation of 2D digital documentation. Therefore, almost every fifth article was considered to have a general scope of 17.10%. AI now has a significant impact even on dental education, at 5.63%. With its interdisciplinary penetration across all fields, AI seems determined to reorganize dentistry as we know it.

## 5. Conclusions

This research confirms that the current use of artificial intelligence in dentistry is concentrated mainly around the evaluation of digital diagnostic methods, especially radiology, while it is gradually penetrating all parts of the dental profession.

Trends in AI implementation in dentistry are currently strongest in the fields of dental radiology and orthodontics.

In comparison to the two previous AI booms, the power of the current wave is gaining unprecedented momentum, with about 35% growth every year since 2017. This growth is not only the result of the acceleration caused by pandemic, but is also powered by the shift towards 3D/4D advanced diagnostics and big data availability.

## Figures and Tables

**Table 1:** Review protocol, compliant with PRISMA-P (Preferred Reporting Items for Systematic review Protocols ^1^.

Methods	Item No.	Checklist Item
Eligibility criteria	8	Only original articles and reviews in English covering dental topics with objective AI utilization were eligible. On 19 April 2022, PubMed, Scopus, Web of Science—Core Collection, and Google Scholar were queried. The focus for the 1st objective was on publications in the years from 1 January 2011 until 31 December 2021. The focus for the 2nd objective was on publications in the years from January 2021 until 19 April 2022. Only published publications were considered. Only publications truly implementing AI (including subfields) in the context of a dental topic or dental specialization were eligible. Respected subfields of AI were: (#1) Machine Learning, (#2) Deep learning, (#3) Neural Networks, (#4) Cognitive Computing, (#5) Natural Language Processing, (#6) Computer Vision. Study design analysis: Quantitative assessment of dental AI publications 2011–2021. Qualitative assessment of the current literature from 2021 until present. This is not a Cochrane review.
Information sources	9	Only the following four electronic databases were queried on 19 April 2022: PubMed Scopus Web of Science—Core Collection Google Scholar No other sources were used (trial registers or other grey literature sources).
Search strategy	10	PubMed, web search: https://pubmed.ncbi.nlm.nih.gov/advanced/ software: Harzing’s Publish or Perish for batch export, with API key Query: *“artificial intelligence”AND(dental OR dentistry OR tooth OR teeth OR dentofacial OR maxillofacial OR orofacial OR orthodontic OR endodontic OR periodontal OR prosthodontic)* *Type: Articles and reviews in English* *Relevant publication dates for the 1st objective: 1 January 2011–31 December 2021. Relevant publication dates for the 2nd objective: 2021–19 April 2022,* Scopus, web search: https://www.scopus.com/search/form.uri?display=advanced software: Harzing’s Publish or Perish for batch export, with API key Query: *“artificial intelligence”AND(dental OR dentistry OR tooth OR teeth OR dentofacial OR maxillofacial OR orofacial OR orthodontic OR endodontic OR periodontal OR prosthodontic)* *Limited to subject area: Dentistry* *Type: Articles and reviews in English* *Relevant publication dates for the 1st objective: 1 January 2011–31 December 2021. Relevant publication dates for the 2nd objective: 2021–19 April 2022* Web of Science—Core Collection web search: https://www.webofscience.com/wos/woscc/advanced-search software: Publish or Perish for batch export, IP based access with login/password Query: *“artificial intelligence”AND(dental OR dentistry OR tooth OR teeth OR dentofacial OR maxillofacial OR orofacial OR orthodontic OR endodontic OR periodontal OR prosthodontic)* *Limited to subject area: Dentistry Oral Surgery Medicine* *Type: Articles or review articles in English* *Relevant publication dates for the 1st objective: 1 January 2011–31 December 2021. Relevant publication dates for the 2nd objective 2021–19 April 2022* Google Scholar, web advanced search: https://scholar.google.com/#d=gs_asd&t=1650796298842 software: Harzing’s Publish or Perish for batch export Query: *“artificial intelligence” AND (dental OR dentistry OR tooth OR teeth OR dentofacial OR maxillofacial OR orofacial OR orthodontics OR endodontics OR periodontics OR prosthodontics) -motor -rotors -gears -”amelogenesis Imperfecta”* Defined Custom year range *Type: All, excluding patents and citations* *Relevant publication dates for the 1st objective: 1 January 2011–31 December 2021. Relevant publication dates for the 2nd objective: 2021–19 April 2022*
Study records:		
Data management	11a	Search and batch export with software: Harzing’s “Publish or Perish” (https://harzing.com/resources/publish-or-perish (accessed on 23 March 2022)). Version for Microsoft Windows: 8.2.3944 (from 23 March 2022), (Windows 11 pro; Microsoft Corp., Redmond, WA, USA) and processed in Microsoft Excel (Excel 365; Microsoft Corp., Redmond, WA, USA). For export and data manipulation, Google Sheets (Alphabet Inc., Mountain View, CA, USA) were also used. This is an online spreadsheet program included as part of the free, web-based Google Docs Editors suite offered by Google.
Selection process	11b	Three independent reviewers conducted each phase of the review.
Data collection process	11c	As the first objective was solely quantitative, the need to extract data from reports was valid only for the second qualitative objective. Publication title and abstract analysis defined the focus of each paper; this was performed separately by three reviewers. Upon the preliminary evaluation of registered papers, 22 dental topics/specializations were defined. Only in cases where an eligible publication was not clearly classifiable from the title and abstract, was a full text analyzed by three independent reviewers. After the reviewers reached a consensus, either the paper’s focus was recorded, or (more frequently) the paper was removed as irrelevant. Only one qualitative marker was assigned to each selected publication for the 2nd objective. In cases of discrepancy between independent reviewers, a consensus had to be reached. The dominant dental topic regarding AI utilization was evaluated, unless the article was equally balanced between two specializations. There were 5 areas that covered all undividable interdisciplinary intersections, which were mostly found between dental radiology, orthodontics, and surgery. For these cases, five border groups had to be defined.
Data items	12	All 22 qualitative variables included five interdisciplinary groups of papers (# 4,5,6,8,9).
Outcomes and prioritization	13	The primary outcome of the 1st objective was the list of selected publications for each year. The primary outcome of the 2nd objective was the list of qualitatively evaluated publications distributed into 22 focus groups/topics. Papers focused on more than two qualitative groups were assigned to “general scope” group. Papers addressing dental education in at least one of the defined topics/fields were assigned separately to the “dental education” group.
Risk of bias in individual studies	14	Each eligible study was evaluated independently by three reviewers. To minimize risk of bias of individual studies, only the studies truly dealing with AI implementation were included. As a number of discrepancies occurred between the three independent reviewers, further tools used to assess the risk of bias in the systematic review were considered. Each initial disagreement was noted.
Data synthesis	15a	The 1st objective provided a list of selected papers. These were grouped according to their publication year and their topics summarized for each year. Percentual increments per year were calculated. The 2nd objective provided a list of selected papers to be distributed into defined focus groups.
	15b	For simplicity, no combining of data from other studies was planned.
	15c	No other additional analyses were proposed (such as sensitivity or subgroup analyses, or meta-regression).
	15d	If quantitative synthesis was not appropriate, a systematic narrative synthesis was provided with information presented in the text and tables to summarize and explain the impact of included studies.
Meta-bias(es)	16	The weight of each study included was equivalent. Overall, the selective reporting of study results (and the failure to publish small or nonsignificant results) leads to the overestimation of intervention effects in systematic reviews, a phenomenon called meta-bias.
Confidence in cumulative evidence	17	For the simplicity of this review design, only a “summary of findings” tables are included with a summary of the amount of evidence, in accordance with the GRADE framework (GRADE Working Group, 2004; Guyatt et al. [72]) which combines considerations of risk of bias, directness, heterogeneity, precision, and publication bias [72].

^1^ Acknowledgement to Statement paper by Moher et al. [71] and Explanation and Elaboration paper by Shamseer et al. [70].

**Table 2:** Results of synthesis of annual publication counts of AI-focused dental publications from various electronic databases, contrasting registered and finally included series of publications with percentual annual increase evaluation.

Year	PubMed	Scopus ^1^	WOS ^1^	Google Scholar	Registered	Included	Increase
2011	4	18	0	107	129	55	
2012	2	17	0	130	149	60	+9.09%
2013	14	24	2	136	166	66	+10.00%
2014	11	14	0	133	168	68	+3.03%
2015	11	27	0	144	182	72	+5.88%
2016	13	24	3	189	229	82	+13.89%
2017	5	23	1	252	281	101	+23.17%
2018	37	34	5	310	386	139	+37.62%
2019	83	59	24	435	601	201	+44.60%
2020	229	107	60	460	856	291	+44.78%
2021	407	250	119	490	1266	362	+24.40%

^1^ Limited to Dentistry.

**Table 3:** Results of synthesis for AI-focused dental papers (1 January 2021–19 April 2022) included for qualitative assessment.

Year.	PubMed	Scopus ^1^	WOS ^1^	Google Scholar	Registered	Included
2022	39	85	23	304	451	135
2021	407	250	119	490	1266	362

^1^ Limited to Dentistry.

**Table 4:** Results of synthesis with qualitative variables (22) for classification of included recent studies.

#	Focus in (Topic/Specialty)	Amount	Percentage
1	GENERAL SCOPE ON DENTISTRY	85	17.10%
2	EDUCATION IN DENTISTRY	28	5.63%
3	RADIOLOGY AND IMAGING DIAGNOSTICS	101	20.32%
4	*RADIOLOGY—ORTHODONTICS* ^1^	6	1.21%
5	*RADIOLOGY—RESTORATIVE DENTISTRY* ^1^	16	3.22%
6	*RADIOLOGY—SURGERY* ^1^	8	1.61%
7	ORTHODONTICS	72	14.49%
8	*ORTHODONTICS—FORENSICS* ^1^	10	2.01%
9	*ORTHODONTICS—SURGERY* ^1^	9	1.81%
10	SURGERY (MAXILLOFACIAL)	17	3.42%
11	CANCER DIAGNOSTICS	19	3.82%
12	DENTAL IMPLANTS	23	4.63%
13	RESTORATIVE DENTISTRY incl. CARIES DIAG.	39	7.85%
14	RESTORATIVE PEDIATRIC DENTISTRY	7	1.41%
15	PERIODONTOLOGY	12	2.41%
16	ENDODONTICS	14	2.82%
17	FORENSIC DENTISTRY	11	2.21%
18	PROSTHODONTICS	8	1.61%
19	DSD	5	1.01%
20	REGENERATIVE DENTISTRY	3	0.60%
21	AIRWAY	2	0.40%
22	DENTAL MATERIAL RESEARCH	2	0.40%

^1^ Five interdisciplinary “focus groups” for papers with focus balanced between two classifications (# 4,5,6,8,9).

**Table 5:** The most impactful AI publications in dentistry.

Title	Author	Journal	Year
Detection and diagnosis of dental caries using a deep learning-based convolutional neural network algorithm [76]	Lee, Jae-Hong; et al.	JOURNAL OF DENTISTRY	2018
Diagnosis and prediction of periodontally compromised teeth using a deep learning-based convolutional neural network algorithm [77]	Lee, Jae-Hong; et al.	JOURNAL OF PERIODONTAL AND IMPLANT SCIENCE	2018
Deep Learning for the Radiographic Detection of Apical Lesions [78]	Ekert, Thomas; et al.	JOURNAL OF ENDODONTICS	2019
Convolutional neural networks for dental image diagnostics: A scoping review [79]	Schwendicke, Falk; et al.	JOURNAL OF DENTISTRY	2019
Artificial Intelligence in Dentistry: Chances and Challenges [25]	Schwendicke, F.; et al.	JOURNAL OF DENTAL RESEARCH	2020
An overview of deep learning in the field of dentistry [80]	Hwang, Jae-Joon; et al.	IMAGING SCIENCE IN DENTISTRY	2019
Deep learning in medical image analysis: A third eye for doctors [81]	Fourcade, A.; Khonsari, R. H.	JOURNAL OF STOMATOLOGY ORAL AND MAXILLOFACIAL SURGERY	2019
A deep-learning artificial intelligence system for assessment of root morphology of the mandibular first molar on panoramic radiography [82]	Hiraiwa, Teruhiko; et al.	DENTOMAXILLOFACIAL RADIOLOGY	2019
Deep-learning classification using convolutional neural network for evaluation of maxillary sinusitis on panoramic radiography [83]	Murata, Makoto; et al.	ORAL RADIOLOGY	2019
Staging and grading of oral squamous cell carcinoma: An update [84]	Almangush, Alhadi; et al.	ORAL ONCOLOGY	2020

## Data Availability

We fully adhere to the Data Availability Statements in the section “MDPI Research Data Policies” at https://www.mdpi.com/ethics (accessed on 24 April 2022). Data are available in a publicly accessible repository. The data presented in this study are openly available in https://docs.google.com/spreadsheets/d/16p8OnvTfoQLBp4fUcfxlhSrZ4Ow8di4p3qScGncGuw8/edit?usp=sharing (accessed on 6 June 2022). Raw data is publicly available together with the PRISMA2020 abstract checklist and the PRISMA2020 systematic review checklist, and the PRISMA2020-compliant protocol of systematic review is published and available online. The systematic review protocol is registered and available online in the Registry of Systematic Reviews/Meta-Analyses, reg.number: reviewregistry1349 and in the International Prospective Register of Systematic Reviews—PROSPERO as “CRD42022327520”.

## Supplementary Materials

The following supporting information can be downloaded at: https://docs.google.com/spreadsheets/d/16p8OnvTfoQLBp4fUcfxlhSrZ4Ow8di4p3qScGncGuw8/edit?usp=sharing.
